# Supplementary material for: Design and synthesis of broadband absorption covalent organic framework for efficient artificial photocatalytic amine coupling
Source: Nat Commun. 2024 Jun 7;15:4856. doi: 10.1038/s41467-024-49036-z (PMC11161580; doi:10.1038/s41467-024-49036-z)
Supplement: Supplementary file 1 — Supplementary Information [file 41467_2024_49036_MOESM1_ESM.docx]

Supplementary Information

**Design and Synthesis of Broadband Absorption Covalent Organic Framework for Efficient Artificial Photocatalytic Amine Coupling**

Yuanding Fang^#^, Youxing Liu^#^, Haojie Huang, Jianzhe Sun, Jiaxing Hong, Fan Zhang, Xiaofang Wei, Wenqiang Gao, Mingchao Shao, Yunlong Guo*, Qingxin Tang* and Yunqi Liu*

**Supplementary Note**

The 1,3,6,8-tetrakis(4-aminophenyl)pyrene, *P*-toluene sulfonic acid, diisopropyl malonat, 5-Cyanothiophene-2-carboxaldehyde, 1,2-Dichlorobenzene,Mesitylene, ethylene glycol, t-amyl alcohol, ferric chloride, toluene,5-Cyanothiophene-2-carboxaldehyde, and Potassium tert-butoxide were were purchased from Energy Chemical Co., Ltd, Shanghai Macklin Biochemical Technology Co., Ltd, Aladdin Biochemical Technology Co. Ltd., and Shanghai Bide Pharmaceutical Technology Co., Ltd. All reagents were utilized without purification.

Synthesis of the TpDPP and DPP

 **Supplementary Fig. 1.** Synthesis routes for TpDPP (**5**).

The synthetic procedure **1**‒**3** was according to the literature^1^.

Synthesis of 3,6-bis(5-(1,3-dioxolan-2-yl)thiophen-2-yl)-2,5-bis(2-ethylhexyl)-2,5-dihydropyrrolo[3,4-c]pyrrole-1,4-dione (**4**): A mixture of **3** (5.0 g, 11.5 mmol), potassium tert-butoxide (2.77 g, 24.71 mmol), and dry DMF (140 mL) was heated to 110 °C for 2 h. Then, 3-(bromomethyl) heptane (11.67 g, 85 mmol) was added slowly at 90 °C, and stirred overnight at 110 °C overnight. After cooled to room temperature, and pour the mixture into the ice. Dichloromethane (DCM) was added, and then the mixture was washed with water. The organic layer was dried with anhydrous Na_2_SO_4_, and the solvent was removed under reduced pressure. The product was purified by column chromatography with DCM and ethylacetate (V/V = 10/1) as eluent to give **4** as dark purple solid (3.07 g, 40%).

Synthesis of 5,5'-(2,5-bis(2-ethylhexyl)-3,6-dioxo-2,3,5,6-tetrahydropyrrolo[3,4-c] pyrrole-1,4-diyl)bis (thiophene-2-carbaldehyde) (**5**): **4** (2.0 g, 3.4 mmol) was dissolved in a mixture of THF (40 mL) and HCl (2 M, 50 mL) and the reaction mixture was stirred at 60 °C for 2 h. After cooled to room temperature, DCM (50 mL) was added, and the mixture was washed with water. The organic layer was dried with anhydrous Na_2_SO_4_ and the solvent was removed under reduced pressure. The crude product was purified by column chromatography with DCM and ethylacetate (V/V = 10/1) as eluent to give **5** as a dark purple powder (1.78 g, 90%).

The synthetic procedure DPP was according to the literature^2^ and the alkyl substitution reaction experimental procedure are the same as **4**.

NMR of the TpDPP and DPP

 **Supplementary Fig. 2.** The ^1^H spectra of TpDPP.

**TpDPP**: Dark purple solid; **^1^H NMR** (400 MHz, CDCl_3_): δ 10.03 (s, 2H, CHO), 9.03 (d, *J* = 4.1 Hz, 2H, H_Ar_), 7.87 (d, *J* = 4.2 Hz, 2H, H_Ar_), 4.30-3.86 (m, 4H, CH_2_), 1.82 (q, *J* = 6.1 Hz, 2H, CH), 1.43-1.13 (m, 16H, CH_2_), 0.87 (dt, *J* = 13.1, 7.2 Hz, 12H, CH_3_).

 **Supplementary Fig. 3.** The ^13^C NMR spectra of TpDPP.

**TpDPP**: **^13^C NMR** (400 MHz, CDCl_3_): δ 182.9 (HC=O), 161.5 (C=O), 146.7 (C_Ar_), 140.7 (C_Ar_), 136.9 (C), 111.0 (C_Ar_), 129.4 (C_Ar_), 111.1 (C), 46.3 (CH_2_), 39.4 (CH), 30.2 (CH_2_), 28.4 (CH_2_), 23.6 (CH_2_), 23.1 (CH_2_), 14.1 (CH_3_), 10.6 (CH_3_). HRMS (MALDI^+^) m/z calcd. For C_32_H_40_N_2_O_4_S_2_^+^) 581.2430, found 581.2499.

 **Supplementary Fig. 4.** The ^1^H NMR spectra of DPP.

**DPP**: Red solid; **^1^H NMR** (400 MHz, CDCl_3_) δ 10.08 (t, *J* = 2.6 Hz, 2H, CHO), 8.14-7.83 (m, 8H, H_Ar_), 3.74 (dd, *J* = 7.0, 3.2 Hz, 4H, CH_2_), 1.58 (s, 1H, CH), 1.43 (s, 3H, CH_3_), 1.10 (d, *J* = 14.7 Hz, 16H, CH_2_), 0.89-0.54 (m, 12H, CH_3_).

+

 **Supplementary Fig. 5.** The ^13^C NMR spectra of DPP.

**DPP**: **^13^C NMR** (400 MHz, CDCl_3_): δ 191.3 (HC=O), 162.4 (C=O), 147.9 (C_Ar_), 137.7 (C), 133.8 (C_Ar_), 130.3 (C_Ar_), 129.4 (C_Ar_), 111.1 (C), 45.3 (CH_2_), 38.8 (CH), 30.4 (CH_2_), 28.4 (CH_2_), 23.8 (CH_2_), 22.9 (CH_2_), 14.0 (CH_3_), 10.5 (CH_3_). HRMS (MALDI^+^) m/z calcd. For C_36_H_44_N_2_O_4_^+^) 569.3301, found 569.3373.

Supplemental Figures

**
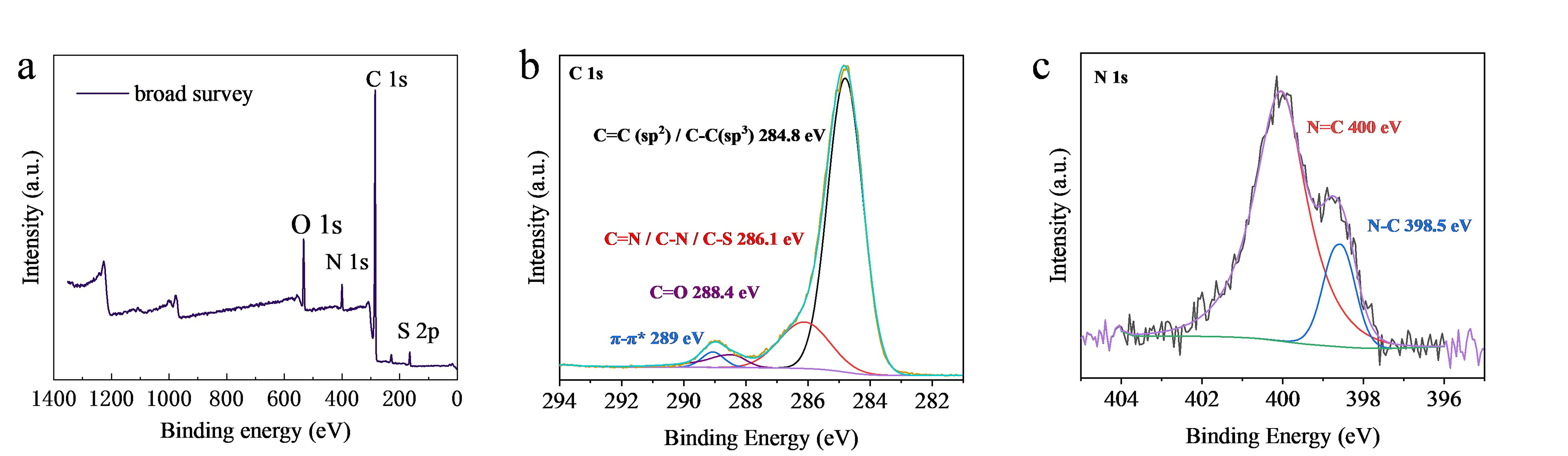
 Supplementary Fig. 6.** (a) XPS profiles including broad survey of TpDPP-Py COFs. (a) C 1s. (b) N 1s XPS spectrum of TpDPP-Py COFs.


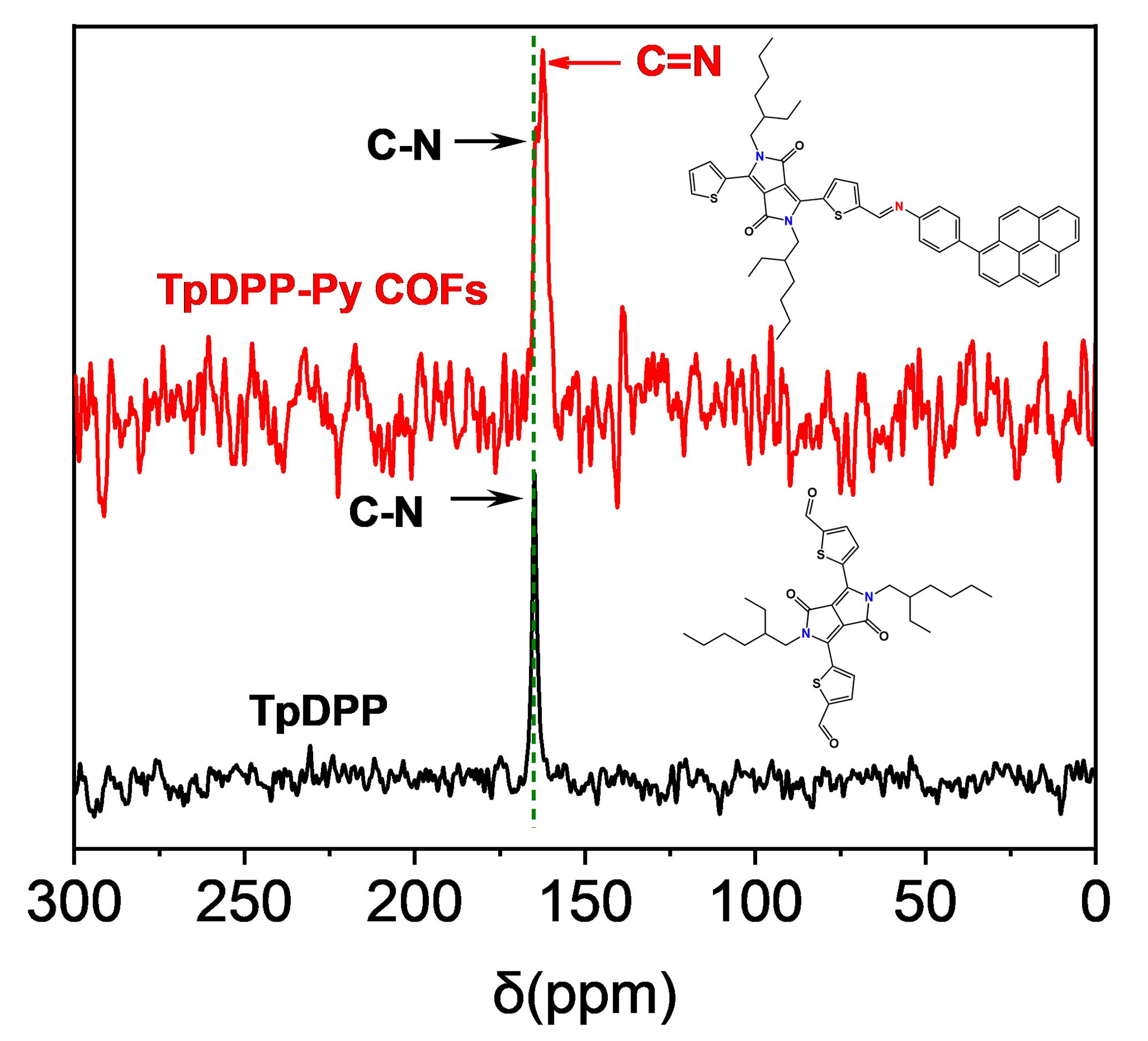


**Supplementary Fig. 7.** ^15^N ssNMR spectroscopy of TpDPP-Py COFs and TpDPP.

**
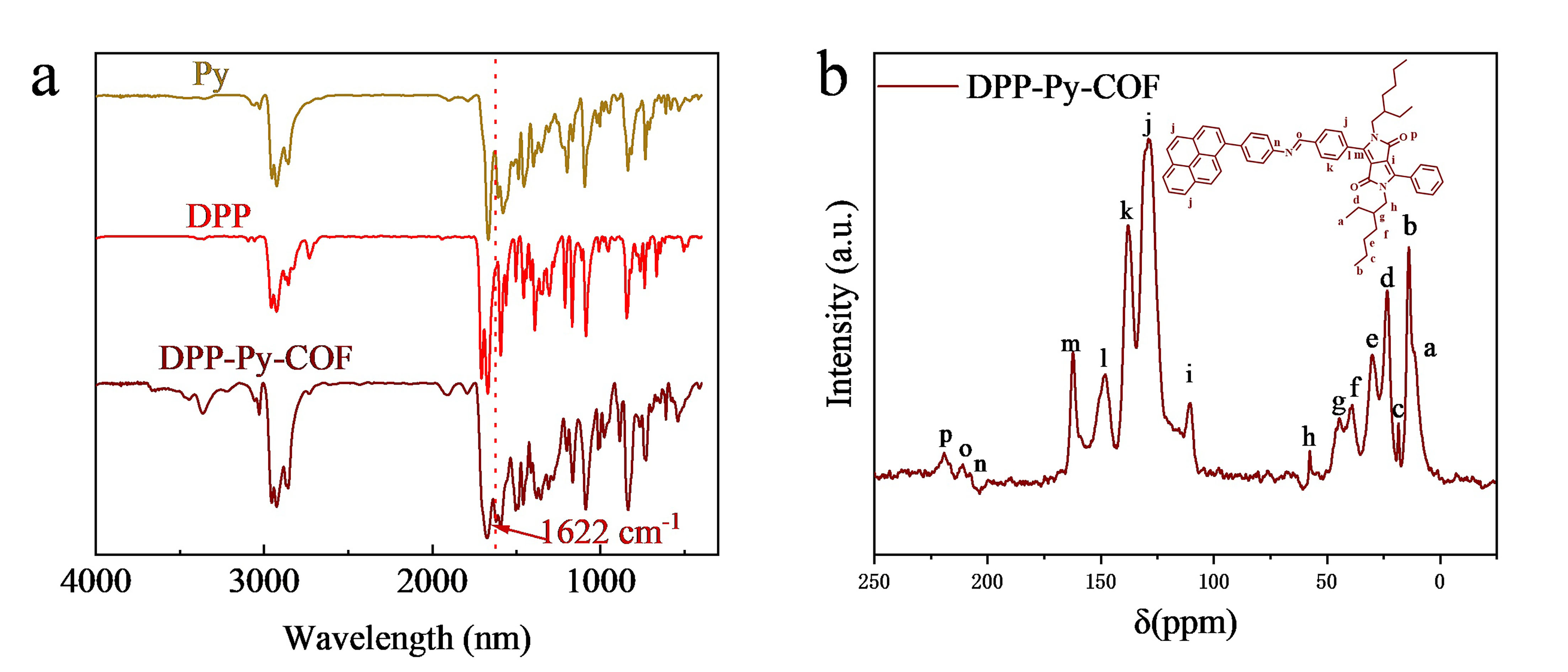
 Supplementary Fig. 8.** (a) FT-IR spectra of DPP, Py and DPP-Py COFs. (b) ^13^C ssNMR spectrum of DPP-Py COFs.


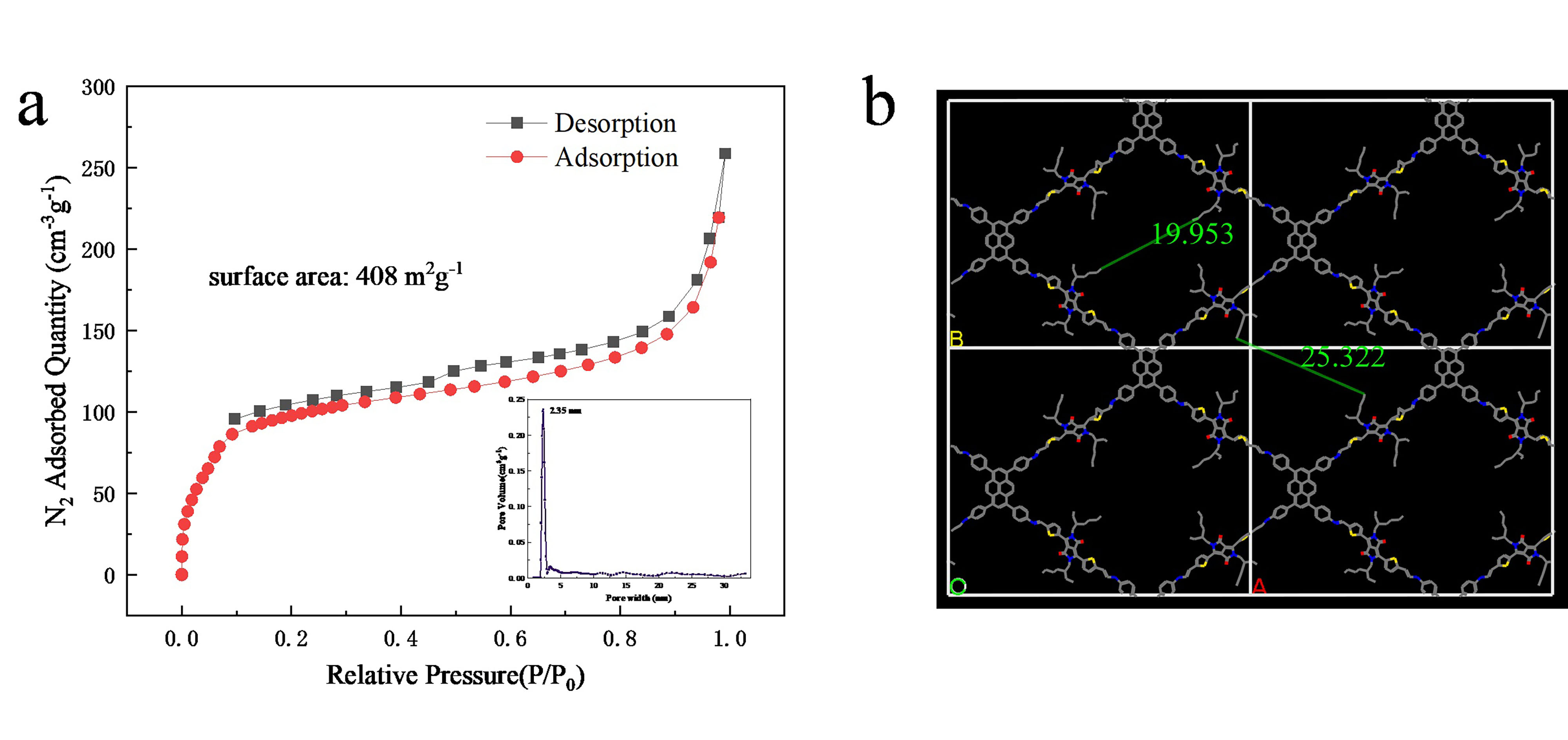


**Supplementary Fig. 9.** (a) Nitrogen sorption isotherm of TpDPP-Py COFs. Full red symbols: adsorption, full black symbols: desorption. (b) Theoretical simulations of TpDPP-Py COFs.


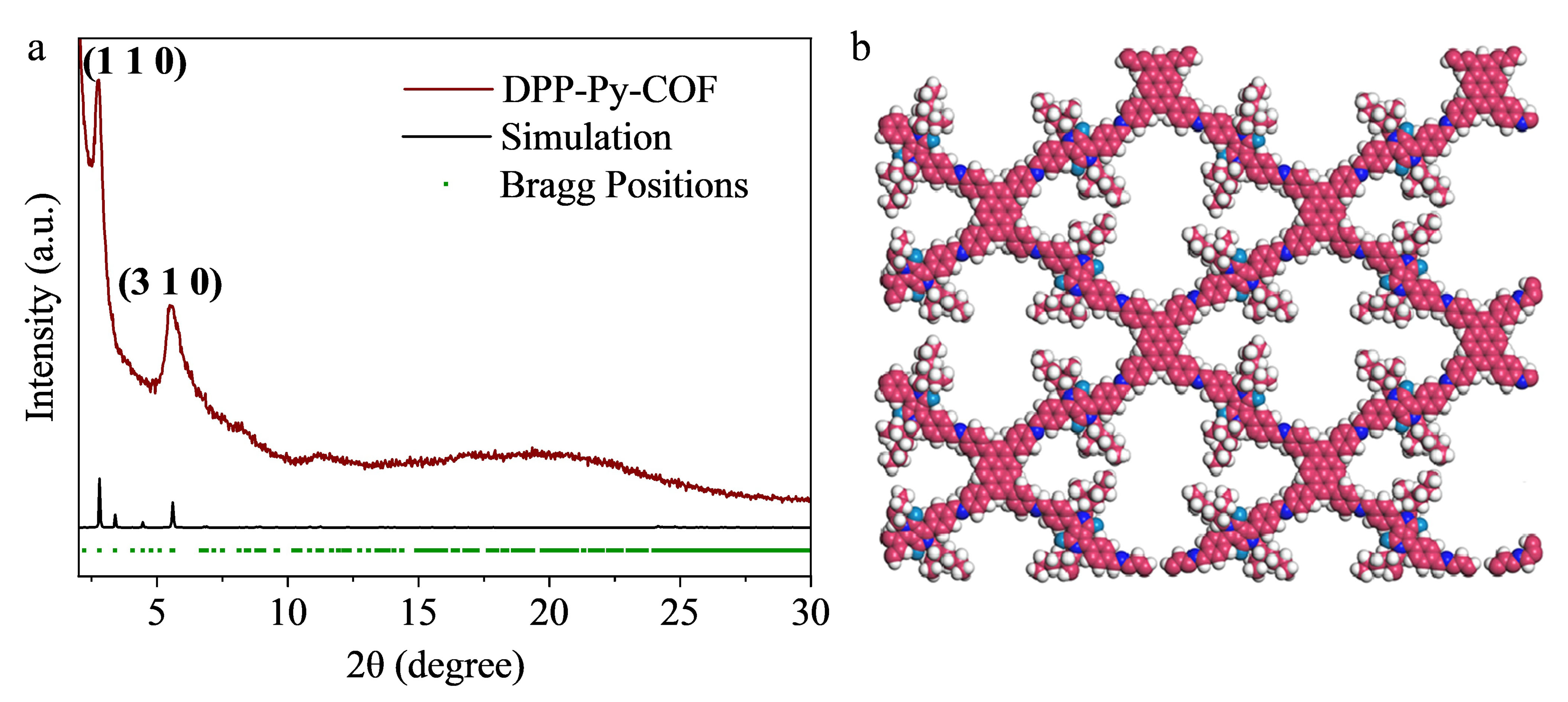


**Supplementary Fig. 10.** (a) Experimentally obtained PXRD powder pattern of DPP-Py COFs (red), simulated pattern with A‒A stacking (black) and the bragg positions (green). (b) Simulated model DPP-Py COFs.


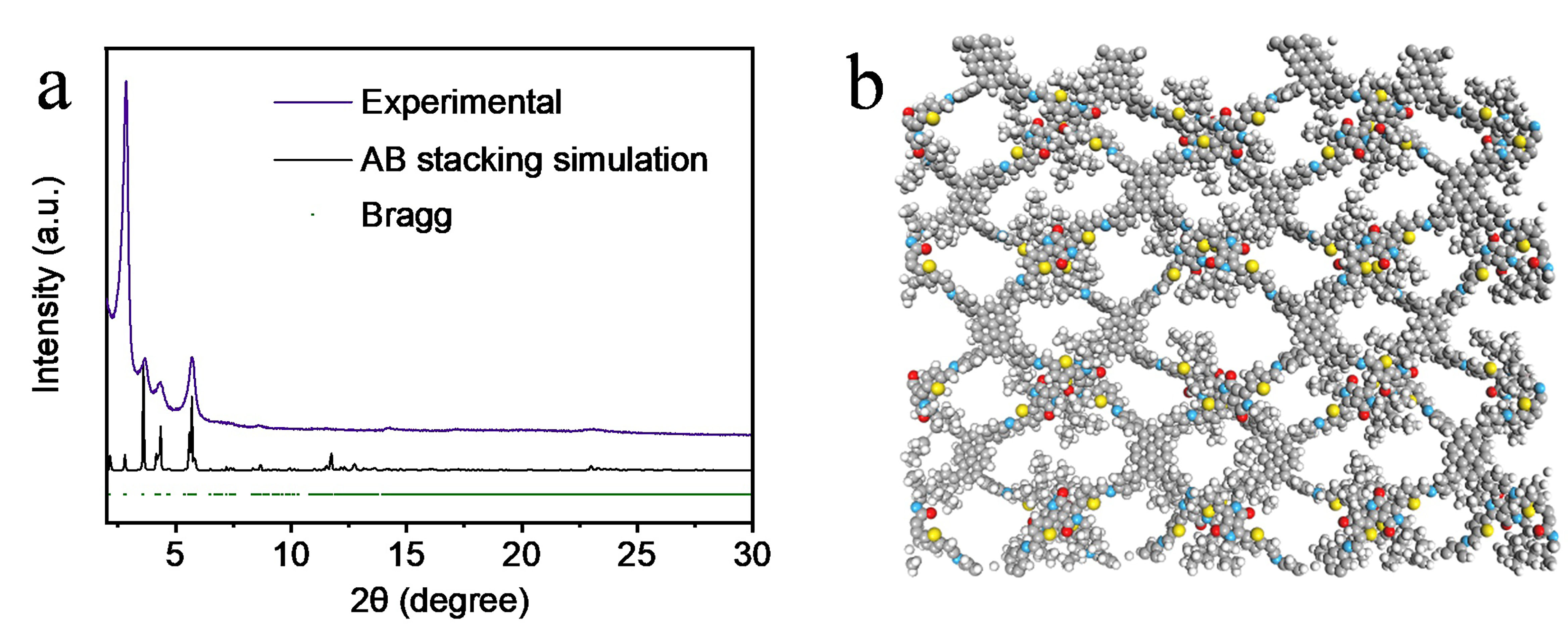


**Supplementary Fig. 11.** (a) Experimentally obtained PXRD powder pattern of TpDPP-Py COFs (purple), simulated pattern with AB stacking (black) and the bragg positions (green). (b) Simulated model TpDPP-Py COFs.


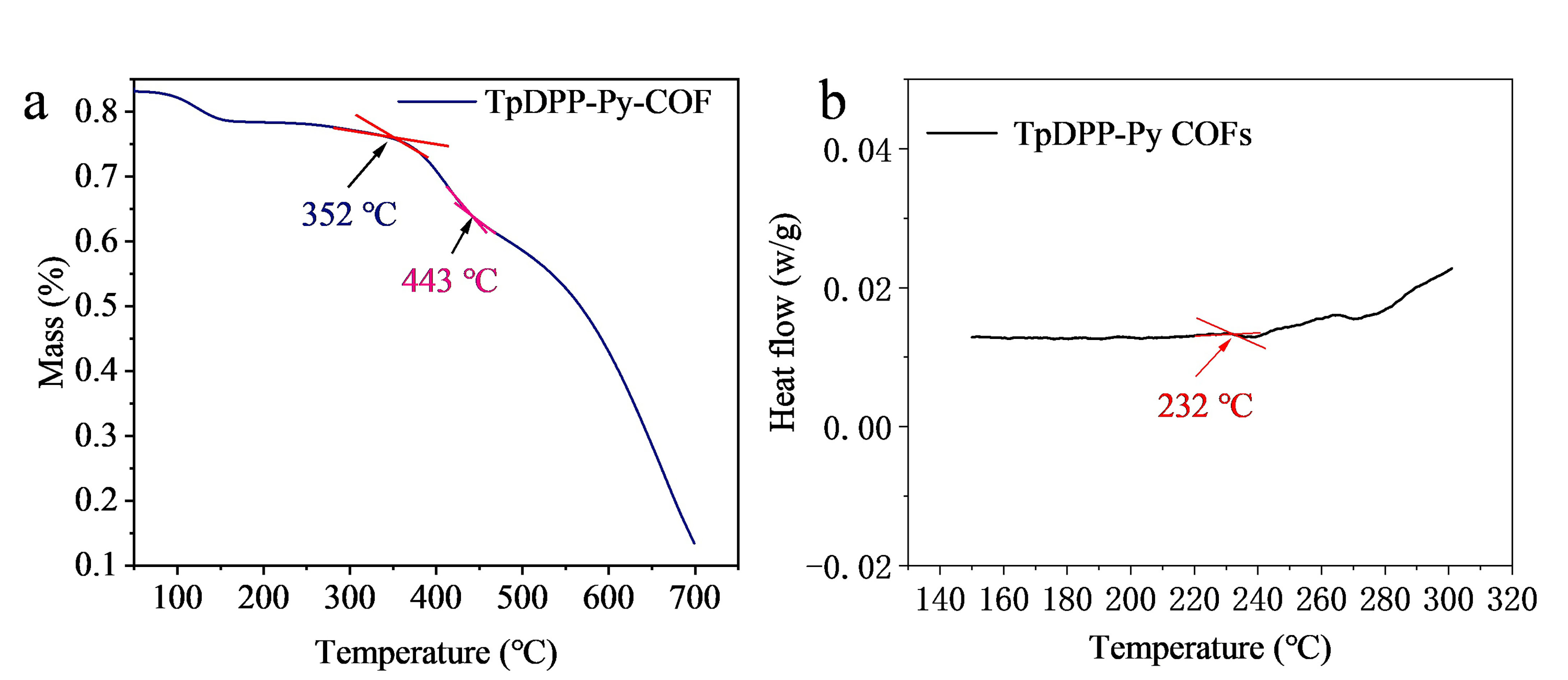


**Supplementary Fig. 12.** (a) Thermogravimetric analysis (TGA) of TpDPP-Py COFs under a nitrogen atmosphere with a heating rate of 10 ℃ min^‒1^. (b) DSC analyses for TpDPP-Py COFs under N_2_.


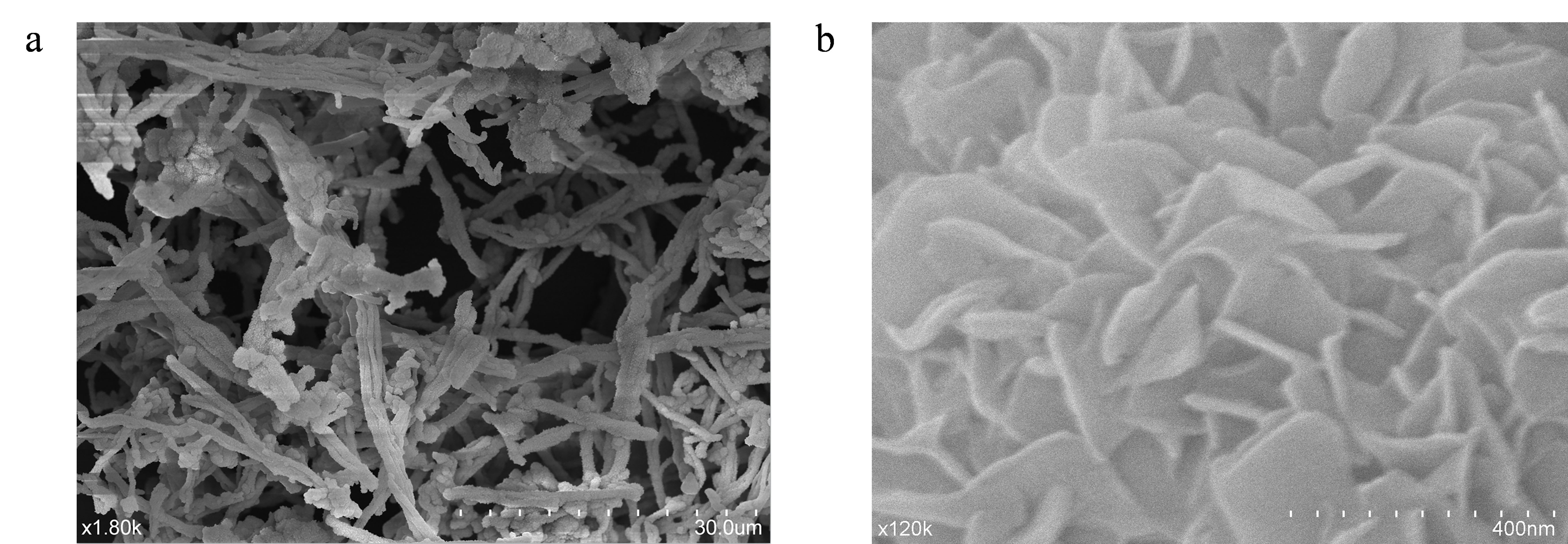


**Supplementary Fig. 13.** (a) SEM images of TpDPP-Py COFs. (b) SEM images of DPP-Py COFs.

Scanning electron microscopy (SEM) was used to investigate the morphology of TpDPP-Py COFs and DPP-Py COFs. TpDPP-Py COFs exhibit a rod-shaped morphology (Supplementary Fig. 13a), while DPP-Py COFs have a sheet morphology (Supplementary Fig. 13b).


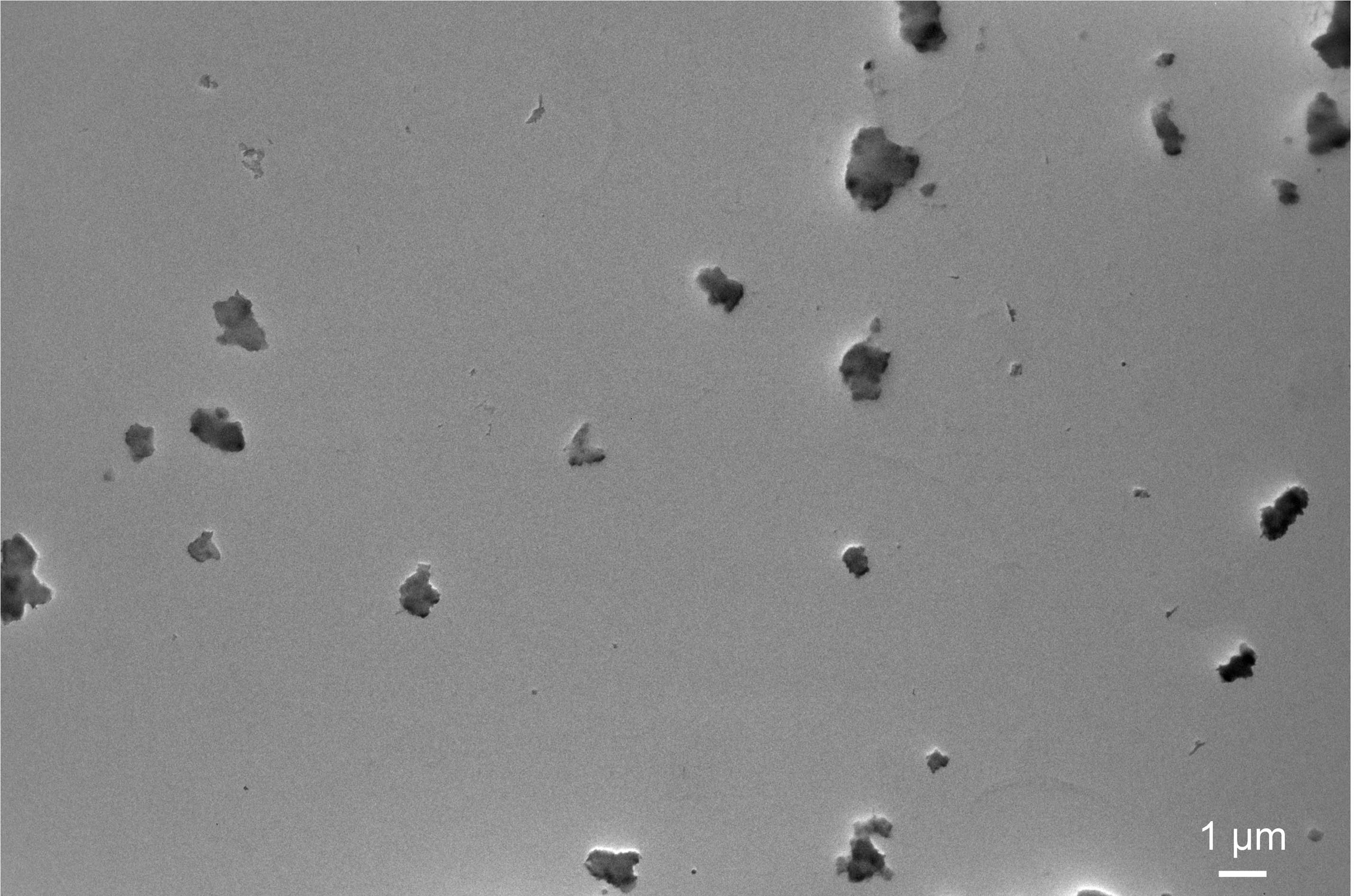


**Supplementary Fig. 14.** THE HR-TEM image of TpDPP Py COFs.

**
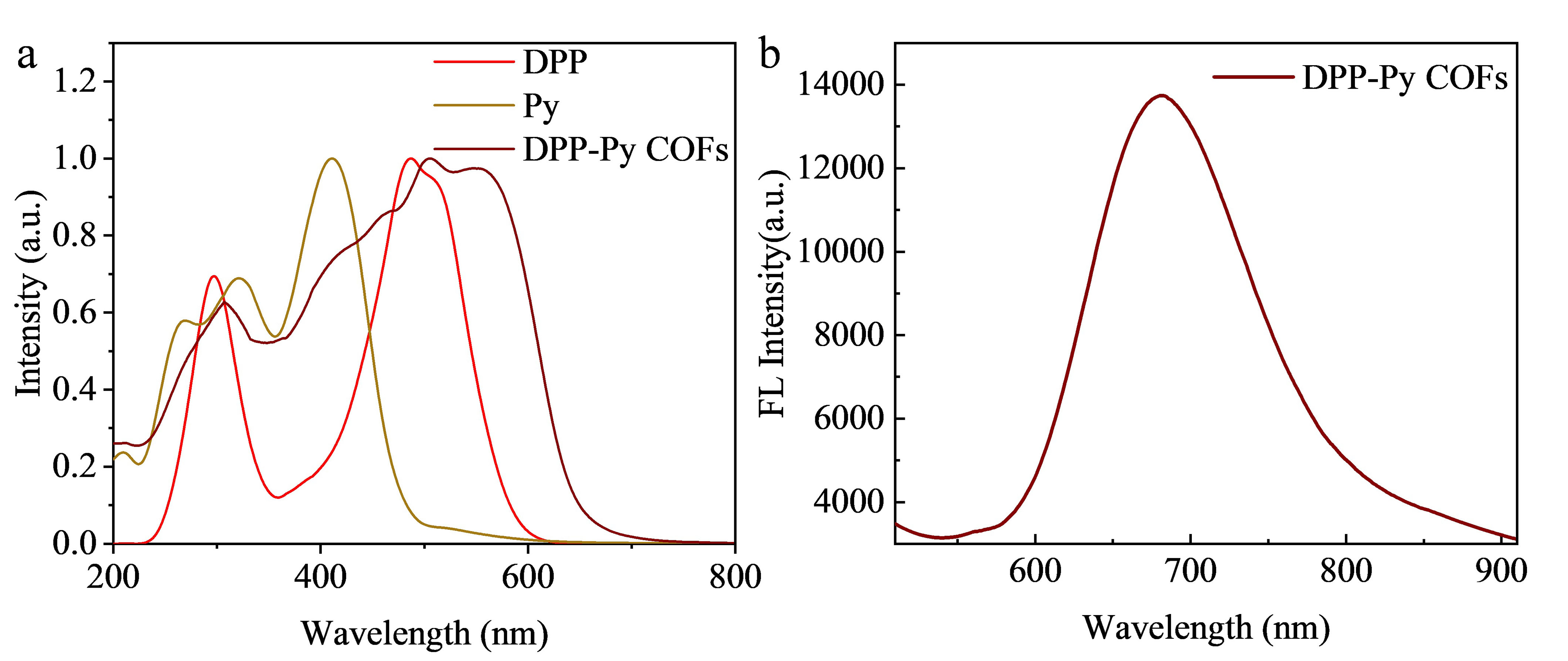
Supplementary Fig. 15.** (a) Kubelka Munk Function UV/Vis Absorption Spectra of DPP-Py COFs, DPP and Py diffuse reflection spectra precursors. (b) Solid-state fluorescence spectrum of DPP-Py COFs (λ_ex_ = 500 nm)


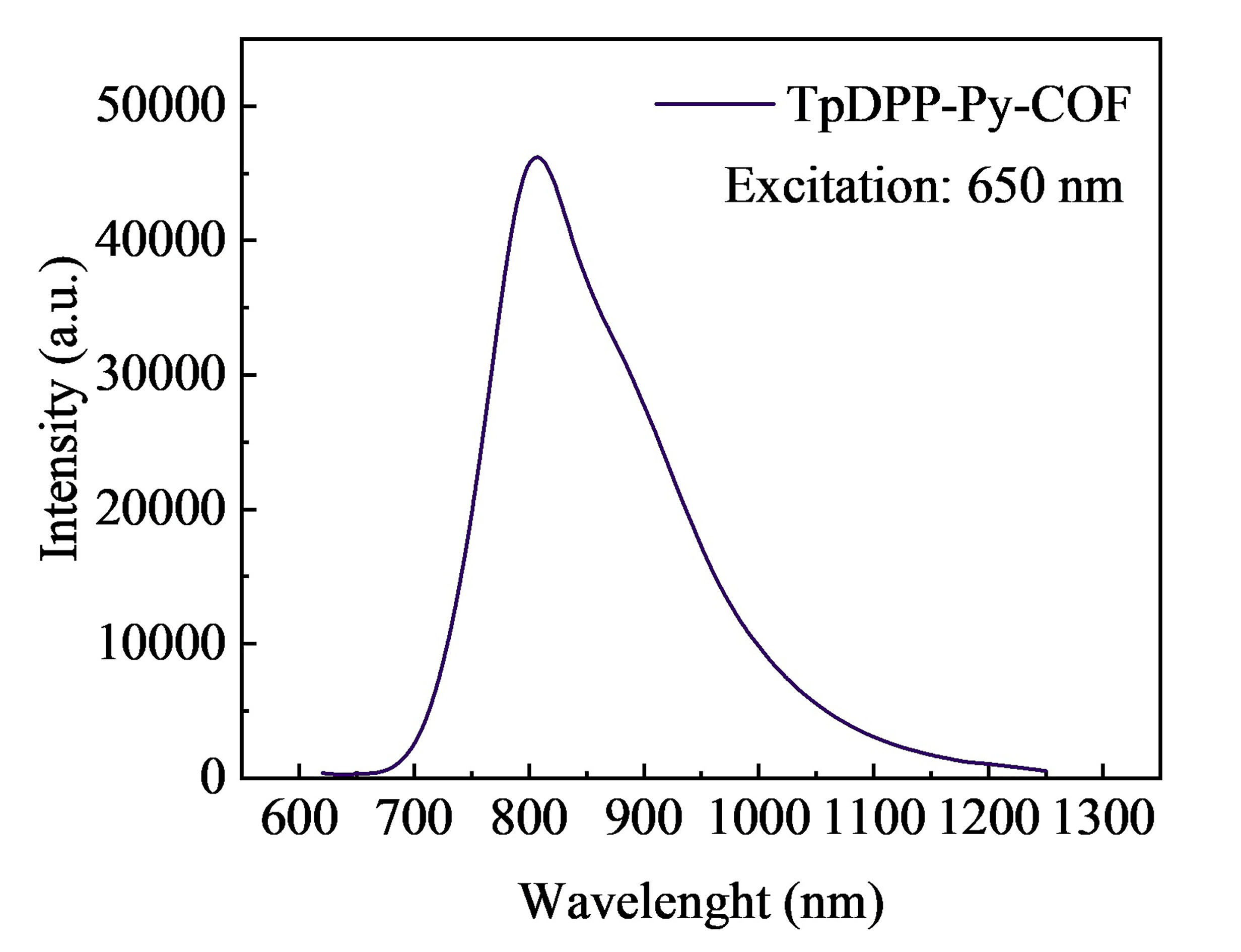


**Supplementary Fig. 16.** The solid-state fluorescence spectrum of TpDPP-Py COFs (λ_ex_ = 650 nm).

Solid-state fluorescence spectrum was carried out to study the fluorescence properties of TpDPP-Py COFs, showing that a strong fluorescence peak appears at 810 nm.

**
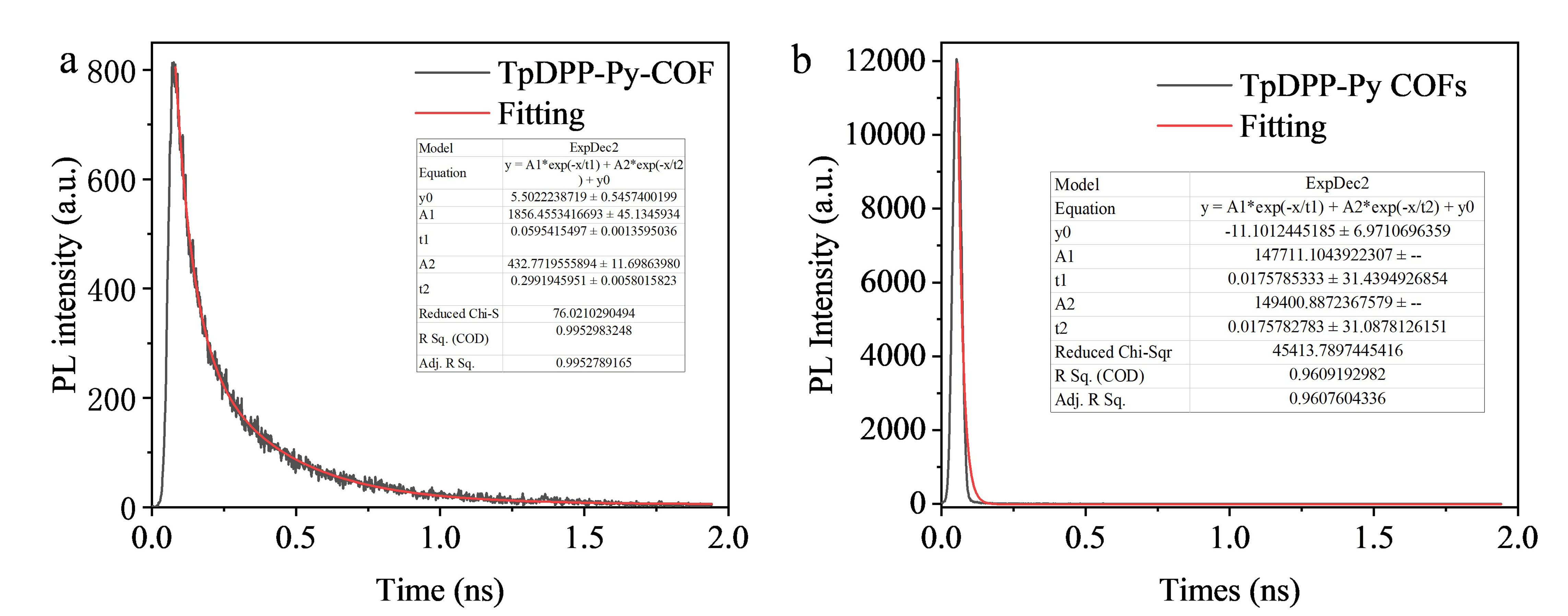
 Supplementary Fig. 17** (a) Time-resolved photoluminescence spectra of TpDPP-Py COFs (λ_ex_ = 1200 nm). (b) Time-resolved photoluminescence spectra of TpDPP-Py COFs (λ_ex_ = 650 nm).


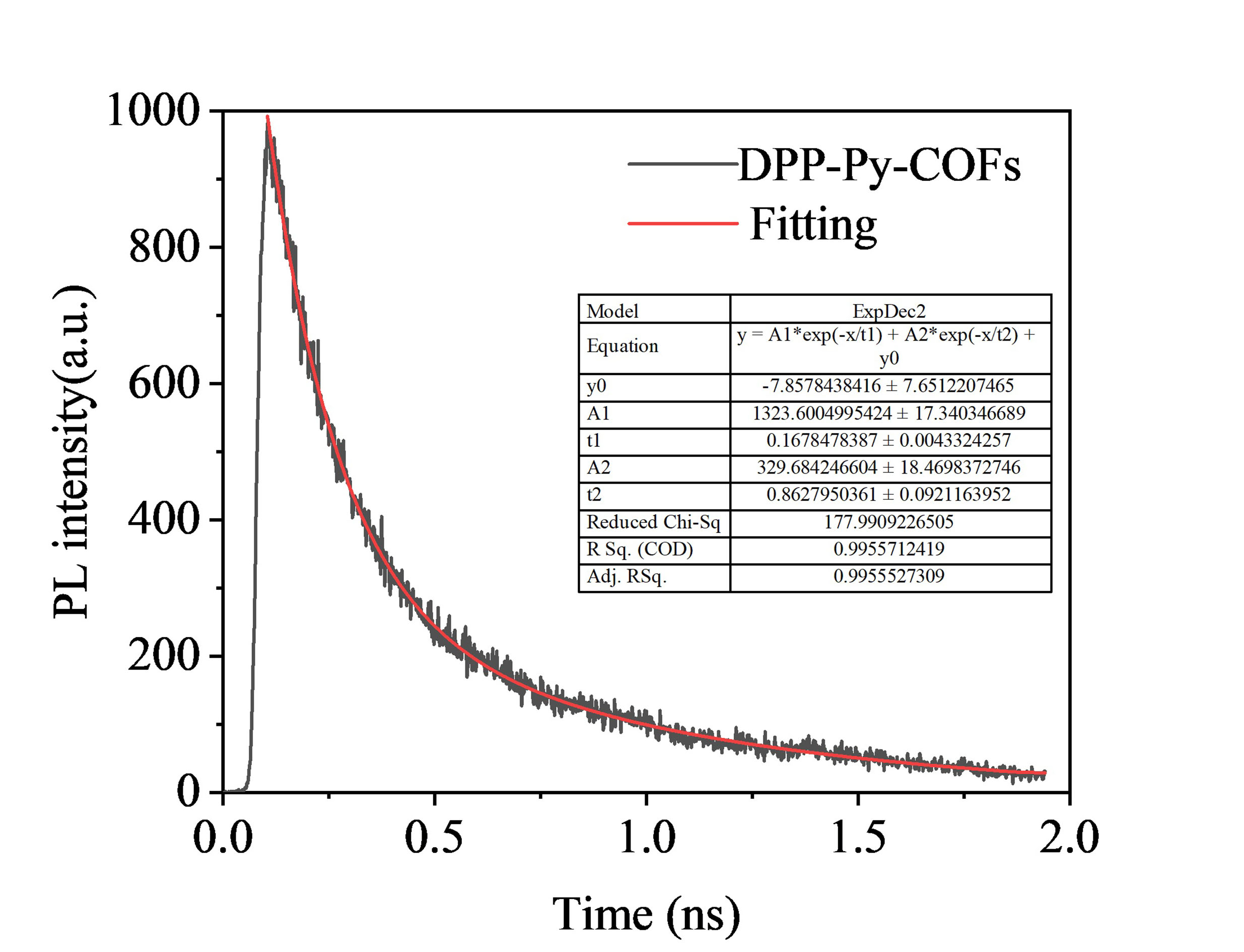


**Supplementary Fig. 18.** Time-resolved photoluminescence spectra of DPP-Py COFs (λ_ex_ = 1200 nm).


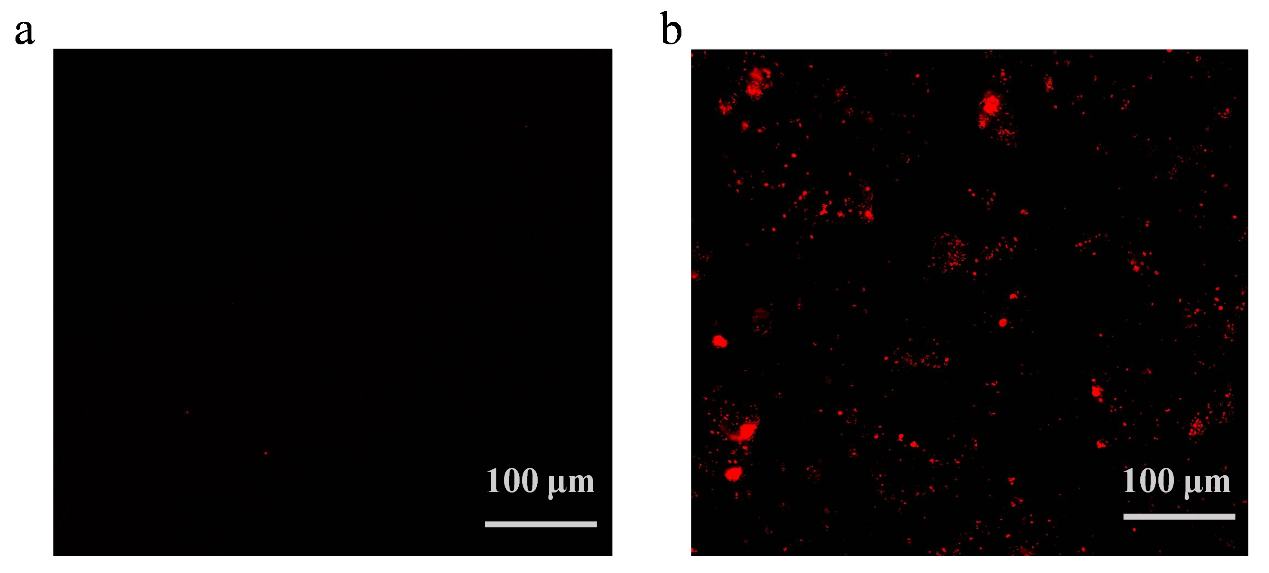


**Supplementary Fig. 19.** (a) Two-photon fluorescence imaging of DPP-Py COFs (λ_ex_ = 1100 nm). (b) Two-photon fluorescence imaging of TpDPP-Py COFs (λ_ex_ = 1100 nm).

**Supplementary Fig. 20.** Gas Chromatogram of BA converted to N-benzylidenebenzylamine over TpDPP-Py COFs.

**Supplementary Fig. 21.** Gas Chromatogram of BA converted to N-benzylidenebenzylamine over TpDPP-Py COFs with 5 batch.


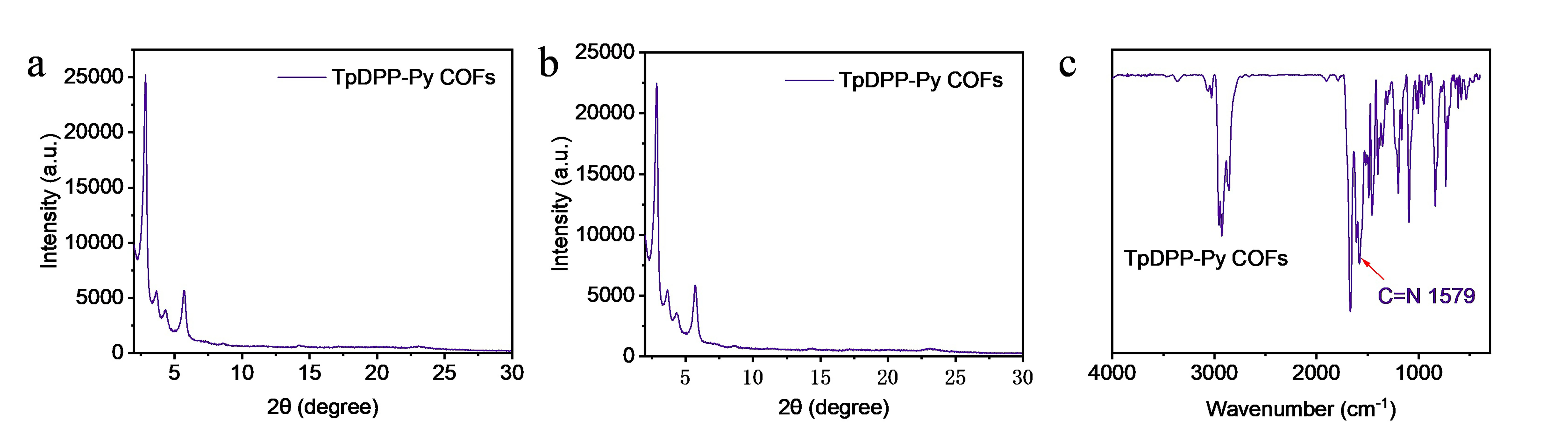


**Supplementary Fig. 22.** (a) XRD pattern of TpDPP Py COFs before photocatalytic reaction. (b) XRD pattern of TpDPP Py COFs after photocatalytic reaction. (c) FT-IR spectra of TpDPP Py COFs after photocatalytic reaction.

**Supplementary Fig. 23.** EPR detection of the ^1^O_2_ trapped by TEMP over DPP-Py COFs under dark and light irradiation.

**Supplementary Fig. 24.** EPR detection of the ^∙^O_2_^–^ anion radical trapped by DMPO over DPP-Py COFs under dark and light irradiation.


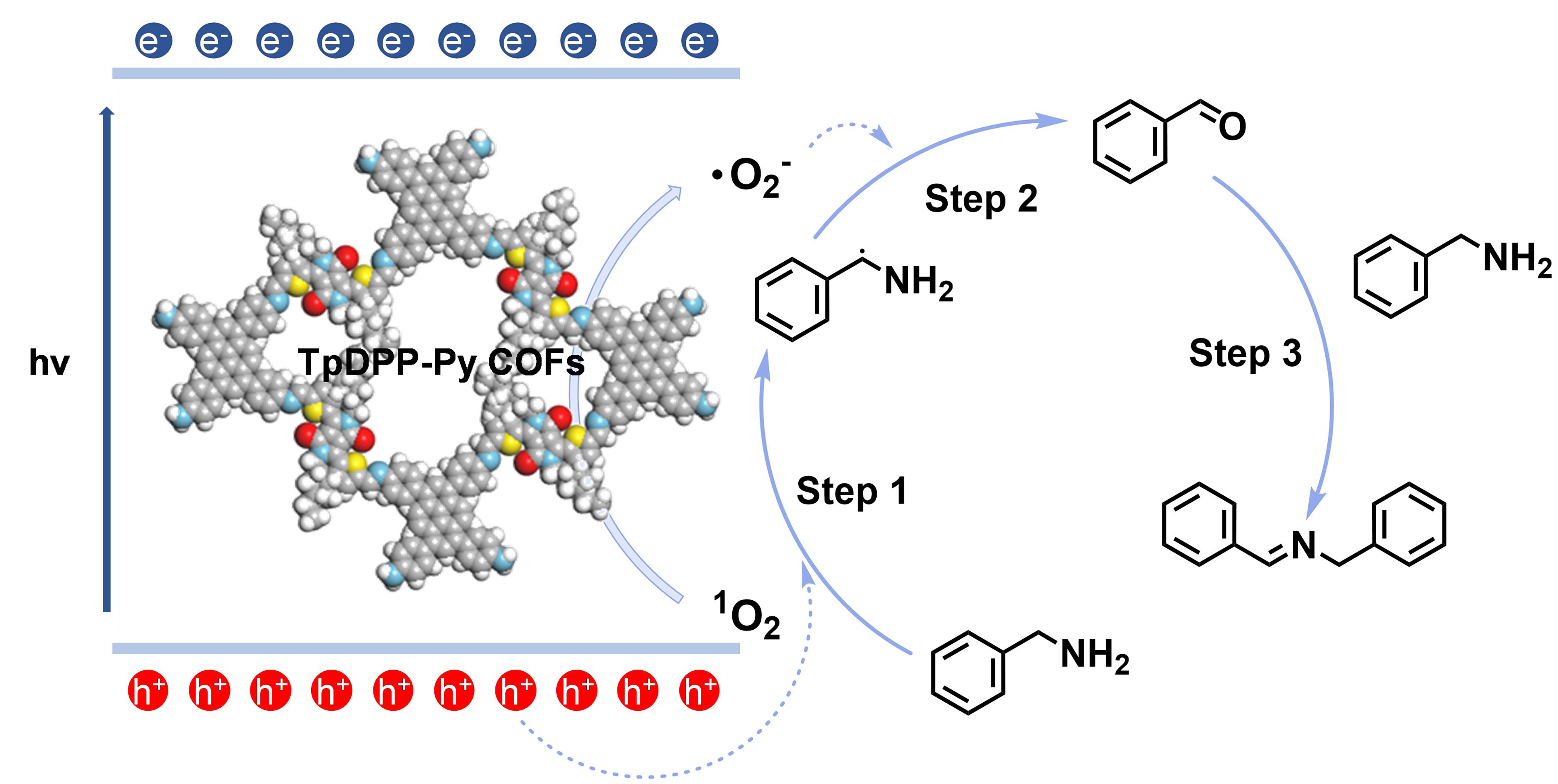


**Supplementary Fig. 25.** Schematic diagram of the mechanism for photocatalytic selective conversion of BA to N-benzylidenebenzylamine.

**Supplementary Table 1.** Atomic coordinates of the AA-stacking mode of TpDPP-Py COFs using DFTB+ method.

| Space group: P1 | | | | |
| --- | --- | --- | --- | --- |
| a = 49.06 Å, b = 40.77 Å and c = 3.88 Å. | | | | |
| α = 89.88°, β = 89.84°and γ = 90.09° | | | | |
|  | Atom | X | Y | Z |
| 1 | C1 | 0.14185 | 0.49628 | 0.30163 |
| 2 | C2 | 0.14282 | 0.46128 | 0.31774 |
| 3 | C3 | 0.16718 | 0.44482 | 0.41274 |
| 4 | C4 | 0.19187 | 0.46362 | 0.44482 |
| 5 | C5 | 0.19213 | 0.49841 | 0.39768 |
| 6 | C6 | 0.16687 | 0.5136 | 0.34499 |
| 7 | C7 | 0.16696 | 0.40986 | 0.47078 |
| 8 | C8 | 0.19155 | 0.39305 | 0.54911 |
| 9 | C9 | 0.21579 | 0.41217 | 0.54845 |
| 10 | C10 | 0.21572 | 0.44619 | 0.52039 |
| 11 | C11 | 0.12013 | 0.44128 | 0.23221 |
| 12 | C12 | 0.11978 | 0.40766 | 0.2975 |
| 13 | C13 | 0.14212 | 0.39125 | 0.44189 |
| 14 | C14 | 0.14064 | 0.35739 | 0.53917 |
| 15 | C15 | 0.16545 | 0.34265 | 0.63219 |
| 16 | C16 | 0.19125 | 0.35856 | 0.61893 |
| 17 | C17 | 0.11779 | 0.51513 | 0.25257 |
| 18 | C18 | 0.21606 | 0.51883 | 0.39226 |
| 19 | C19 | 0.11633 | 0.33746 | 0.5649 |
| 20 | C20 | 0.21449 | 0.33849 | 0.67621 |
| 21 | C21 | 0.11838 | 0.54441 | 0.05352 |
| 22 | C22 | 0.09539 | 0.56416 | 0.01769 |
| 23 | C23 | 0.07014 | 0.555 | 0.16937 |
| 24 | C24 | 0.06897 | 0.52568 | 0.36226 |
| 25 | C25 | 0.09241 | 0.50671 | 0.40982 |
| 26 | C26 | 0.21778 | 0.54955 | 0.56387 |
| 27 | C27 | 0.24211 | 0.56853 | 0.5654 |
| 28 | C28 | 0.26583 | 0.55689 | 0.405 |
| 29 | C29 | 0.26385 | 0.52741 | 0.21562 |
| 30 | C30 | 0.23931 | 0.50939 | 0.20362 |
| 31 | C31 | 0.09135 | 0.35037 | 0.68152 |
| 32 | C32 | 0.06801 | 0.33074 | 0.73461 |
| 33 | C33 | 0.06857 | 0.29657 | 0.67905 |
| 34 | C34 | 0.09269 | 0.28319 | 0.54782 |
| 35 | C35 | 0.11597 | 0.30326 | 0.49082 |
| 36 | C36 | 0.23687 | 0.34819 | 0.88445 |
| 37 | C37 | 0.26044 | 0.32928 | 0.91073 |
| 38 | C38 | 0.26192 | 0.29849 | 0.74907 |
| 39 | C39 | 0.23899 | 0.28703 | 0.56299 |
| 40 | C40 | 0.21607 | 0.30692 | 0.5228 |
| 41 | C41 | 0.39825 | 0.22755 | 0.45404 |
| 42 | C42 | 0.40321 | 0.19671 | 0.59659 |
| 43 | C43 | 0.42981 | 0.18947 | 0.56284 |
| 44 | C44 | 0.44228 | 0.21561 | 0.39467 |
| 45 | C45 | 0.39093 | 0.1704 | 0.76756 |
| 46 | C46 | 0.4349 | 0.15867 | 0.70202 |
| 47 | C47 | 0.3724 | 0.24228 | 0.45131 |
| 48 | C48 | 0.46055 | 0.14374 | 0.71136 |
| 49 | C49 | 0.42884 | 0.26753 | 0.11832 |
| 50 | C50 | 0.43683 | 0.30215 | 0.27312 |
| 51 | C51 | 0.46804 | 0.31226 | 0.25679 |
| 52 | C52 | 0.48995 | 0.28986 | 0.41993 |
| 53 | C53 | 0.51104 | 0.27176 | 0.19598 |
| 54 | C54 | 0.53189 | 0.25054 | 0.38851 |
| 55 | C55 | 0.40471 | 0.1172 | 0.0156 |
| 56 | C56 | 0.38964 | 0.08696 | 0.84374 |
| 57 | C57 | 0.35817 | 0.09333 | 0.8126 |
| 58 | C58 | 0.33857 | 0.06474 | 0.70378 |
| 59 | C59 | 0.30901 | 0.07593 | 0.63386 |
| 60 | C60 | 0.28976 | 0.04746 | 0.52219 |
| 61 | C61 | 0.46435 | 0.11061 | 0.64972 |
| 62 | C62 | 0.49123 | 0.10151 | 0.6688 |
| 63 | C63 | 0.5089 | 0.12781 | 0.74274 |
| 64 | C64 | 0.36767 | 0.27565 | 0.4907 |
| 65 | C65 | 0.34043 | 0.2829 | 0.53544 |
| 66 | C66 | 0.32373 | 0.25491 | 0.53203 |
| 67 | C67 | 0.29439 | 0.25304 | 0.62065 |
| 68 | C68 | 0.53747 | 0.12614 | 0.76357 |
| 69 | C69 | 0.39465 | 0.6481 | 0.47654 |
| 70 | C70 | 0.42051 | 0.64779 | 0.62449 |
| 71 | C71 | 0.43296 | 0.67749 | 0.59691 |
| 72 | C72 | 0.41487 | 0.69766 | 0.42767 |
| 73 | C73 | 0.43819 | 0.62754 | 0.7962 |
| 74 | C74 | 0.45896 | 0.67631 | 0.74331 |
| 75 | C75 | 0.37662 | 0.62075 | 0.45657 |
| 76 | C76 | 0.47787 | 0.70273 | 0.75851 |
| 77 | C77 | 0.3698 | 0.69137 | 0.14573 |
| 78 | C78 | 0.34582 | 0.71114 | 0.31216 |
| 79 | C79 | 0.35402 | 0.74869 | 0.32907 |
| 80 | C80 | 0.33221 | 0.77416 | 0.43058 |
| 81 | C81 | 0.34427 | 0.80938 | 0.47629 |
| 82 | C82 | 0.3227 | 0.83443 | 0.5878 |
| 83 | C83 | 0.48348 | 0.6315 | 0.07188 |
| 84 | C84 | 0.50915 | 0.61467 | 0.90775 |
| 85 | C85 | 0.50849 | 0.57599 | 0.8917 |
| 86 | C86 | 0.48176 | 0.55754 | 0.79083 |
| 87 | C87 | 0.48566 | 0.52089 | 0.69888 |
| 88 | C88 | 0.45867 | 0.50303 | 0.60198 |
| 89 | C89 | 0.47163 | 0.73575 | 0.7996 |
| 90 | C90 | 0.49448 | 0.75634 | 0.79911 |
| 91 | C91 | 0.51851 | 0.7391 | 0.76226 |
| 92 | C92 | 0.38521 | 0.58862 | 0.4119 |
| 93 | C93 | 0.36416 | 0.5658 | 0.40174 |
| 94 | C94 | 0.33887 | 0.58014 | 0.43647 |
| 95 | C95 | 0.31481 | 0.56132 | 0.42353 |
| 96 | C96 | 0.54511 | 0.75197 | 0.75773 |
| 97 | C97 | 0.65188 | 0.99665 | 0.55283 |
| 98 | C98 | 0.65228 | 0.96184 | 0.59156 |
| 99 | C99 | 0.67536 | 0.94359 | 0.48471 |
| 100 | C100 | 0.69901 | 0.96074 | 0.36085 |
| 101 | C101 | 0.70062 | 0.99563 | 0.37342 |
| 102 | C102 | 0.67664 | 0.01255 | 0.4604 |
| 103 | C103 | 0.67472 | 0.90838 | 0.49773 |
| 104 | C104 | 0.69777 | 0.89013 | 0.3903 |
| 105 | C105 | 0.71986 | 0.90784 | 0.24275 |
| 106 | C106 | 0.7203 | 0.94197 | 0.22331 |
| 107 | C107 | 0.63015 | 0.94412 | 0.73717 |
| 108 | C108 | 0.62964 | 0.91 | 0.75343 |
| 109 | C109 | 0.65093 | 0.8912 | 0.61644 |
| 110 | C110 | 0.64901 | 0.85633 | 0.59504 |
| 111 | C111 | 0.67304 | 0.83935 | 0.51264 |
| 112 | C112 | 0.69798 | 0.85528 | 0.42609 |
| 113 | C113 | 0.62813 | 0.01617 | 0.5826 |
| 114 | C114 | 0.7249 | 0.01447 | 0.31279 |
| 115 | C115 | 0.62425 | 0.83789 | 0.6384 |
| 116 | C116 | 0.72169 | 0.8358 | 0.39013 |
| 117 | C117 | 0.62812 | 0.04727 | 0.74747 |
| 118 | C118 | 0.60488 | 0.06718 | 0.74571 |
| 119 | C119 | 0.58074 | 0.05616 | 0.58227 |
| 120 | C120 | 0.58018 | 0.0251 | 0.4264 |
| 121 | C121 | 0.60346 | 0.00576 | 0.42493 |
| 122 | C122 | 0.7502 | 0.0055 | 0.45893 |
| 123 | C123 | 0.77393 | 0.02493 | 0.41454 |
| 124 | C124 | 0.77269 | 0.05476 | 0.2324 |
| 125 | C125 | 0.74788 | 0.06396 | 0.08273 |
| 126 | C126 | 0.72449 | 0.0439 | 0.11825 |
| 127 | C127 | 0.59964 | 0.84854 | 0.48756 |
| 128 | C128 | 0.57507 | 0.83068 | 0.52256 |
| 129 | C129 | 0.57446 | 0.80043 | 0.69645 |
| 130 | C130 | 0.59877 | 0.78904 | 0.84563 |
| 131 | C131 | 0.62312 | 0.80768 | 0.82013 |
| 132 | C132 | 0.72136 | 0.80523 | 0.21603 |
| 133 | C133 | 0.74463 | 0.78551 | 0.20282 |
| 134 | C134 | 0.76929 | 0.79586 | 0.35746 |
| 135 | C135 | 0.77007 | 0.82654 | 0.52469 |
| 136 | C136 | 0.74668 | 0.84572 | 0.54354 |
| 137 | C137 | 0.94186 | 0.64343 | 0.07254 |
| 138 | C138 | 0.93771 | 0.67727 | 0.12602 |
| 139 | C139 | 0.91205 | 0.6836 | 0.21771 |
| 140 | C140 | 0.89951 | 0.65318 | 0.25121 |
| 141 | C141 | 0.95054 | 0.70798 | 0.1272 |
| 142 | C142 | 0.9072 | 0.7173 | 0.24587 |
| 143 | C143 | 0.96835 | 0.63318 | 0.97164 |
| 144 | C144 | 0.88029 | 0.72849 | 0.28699 |
| 145 | C145 | 0.90284 | 0.59593 | 0.13407 |
| 146 | C146 | 0.85819 | 0.54307 | 0.00943 |
| 147 | C147 | 0.93981 | 0.54842 | 0.14675 |
| 148 | C148 | 0.94481 | 0.51215 | 0.25124 |
| 149 | C149 | 0.97543 | 0.5063 | 0.33058 |
| 150 | C150 | 0.98115 | 0.47092 | 0.44871 |
| 151 | C151 | 0.94429 | 0.76598 | 0.21469 |
| 152 | C152 | 0.93358 | 0.80025 | 0.34673 |
| 153 | C153 | 0.90899 | 0.81751 | 0.16478 |
| 154 | C154 | 0.88709 | 0.83693 | 0.38609 |
| 155 | C155 | 0.87511 | 0.87183 | 0.48031 |
| 156 | C156 | 0.84398 | 0.87035 | 0.55182 |
| 157 | C157 | 0.85755 | 0.71302 | 0.13533 |
| 158 | C158 | 0.83297 | 0.72782 | 0.20196 |
| 159 | C159 | 0.83647 | 0.75609 | 0.39601 |
| 160 | C160 | 0.98553 | 0.65214 | 0.75882 |
| 161 | C161 | 0.01271 | 0.64361 | 0.75619 |
| 162 | C162 | 0.01738 | 0.61573 | 0.94988 |
| 163 | C163 | 0.0431 | 0.60176 | 0.98356 |
| 164 | C164 | 0.81599 | 0.77823 | 0.47673 |
| 165 | C165 | 0.54967 | 0.09965 | 0.66761 |
| 166 | C166 | 0.31734 | 0.70333 | 0.14299 |
| 167 | C167 | 0.29041 | 0.70337 | 0.35039 |
| 168 | C168 | 0.95979 | 0.82383 | 0.36831 |
| 169 | C169 | 0.9584 | 0.86069 | 0.46265 |
| 170 | C170 | 0.41998 | 0.3311 | 0.11206 |
| 171 | C171 | 0.41293 | 0.3623 | 0.3162 |
| 172 | C172 | 0.39606 | 0.0515 | 0.97913 |
| 173 | C173 | 0.42606 | 0.04047 | 0.97818 |
| 174 | C174 | 0.91138 | 0.56026 | 0.02352 |
| 175 | C175 | 0.88803 | 0.53375 | 0.08858 |
| 176 | C176 | 0.53706 | 0.62602 | 0.07536 |
| 177 | C177 | 0.5641 | 0.62643 | 0.87049 |
| 178 | C178 | 0.94751 | 0.19423 | 0.77954 |
| 179 | C179 | 0.92098 | 0.20086 | 0.67107 |
| 180 | C180 | 0.9095 | 0.17392 | 0.53021 |
| 181 | C181 | 0.92941 | 0.15032 | 0.53316 |
| 182 | C182 | 0.90269 | 0.22595 | 0.62145 |
| 183 | C183 | 0.88333 | 0.18017 | 0.4076 |
| 184 | C184 | 0.9659 | 0.21901 | 0.89369 |
| 185 | C185 | 0.86552 | 0.15513 | 0.28153 |
| 186 | C186 | 0.97698 | 0.14384 | 0.75428 |
| 187 | C187 | 0.013 | 0.10343 | 0.92639 |
| 188 | C188 | 0.96591 | 0.07498 | 0.78248 |
| 189 | C189 | 0.93556 | 0.067 | 0.88065 |
| 190 | C190 | 0.92634 | 0.03118 | 0.79594 |
| 191 | C191 | 0.89554 | 0.02455 | 0.8557 |
| 192 | C192 | 0.86182 | 0.23628 | 0.28164 |
| 193 | C193 | 0.83671 | 0.25315 | 0.45871 |
| 194 | C194 | 0.82744 | 0.28538 | 0.2717 |
| 195 | C195 | 0.81515 | 0.3139 | 0.48279 |
| 196 | C196 | 0.80587 | 0.34388 | 0.27094 |
| 197 | C197 | 0.79389 | 0.37298 | 0.47048 |
| 198 | C198 | 0.84324 | 0.15888 | 0.06522 |
| 199 | C199 | 0.82573 | 0.13215 | 0.05072 |
| 200 | C200 | 0.83499 | 0.10587 | 0.24241 |
| 201 | C201 | 0.95807 | 0.24642 | 0.0803 |
| 202 | C202 | 0.97827 | 0.2701 | 0.11604 |
| 203 | C203 | 0.0023 | 0.26105 | 0.95641 |
| 204 | C204 | 0.81965 | 0.07715 | 0.30418 |
| 205 | C205 | 0.98093 | 0.10776 | 0.9048 |
| 206 | C206 | 0.02519 | 0.07093 | 0.05933 |
| 207 | C207 | 0.78254 | 0.23523 | 0.47423 |
| 208 | C208 | 0.81287 | 0.2279 | 0.54928 |
| 209 | C209 | 0.02476 | 0.2825 | 0.93548 |
| 210 | N1 | 0.42317 | 0.23874 | 0.33017 |
| 211 | N2 | 0.41014 | 0.14717 | 0.82284 |
| 212 | N3 | 0.28538 | 0.2819 | 0.79259 |
| 213 | N4 | 0.39185 | 0.67954 | 0.35553 |
| 214 | N5 | 0.46165 | 0.64468 | 0.86357 |
| 215 | N6 | 0.2896 | 0.57408 | 0.44057 |
| 216 | N7 | 0.55732 | 0.07272 | 0.55592 |
| 217 | N8 | 0.79355 | 0.07641 | 0.19581 |
| 218 | N9 | 0.55017 | 0.78421 | 0.70646 |
| 219 | N10 | 0.91662 | 0.62861 | 0.14673 |
| 220 | N11 | 0.93247 | 0.73241 | 0.20412 |
| 221 | N12 | 0.79097 | 0.77587 | 0.33171 |
| 222 | N13 | 0.04686 | 0.57231 | 0.13627 |
| 223 | N14 | 0.04698 | 0.27617 | 0.74218 |
| 224 | N15 | 0.95175 | 0.16158 | 0.70584 |
| 225 | N16 | 0.88061 | 0.21377 | 0.44642 |
| 226 | O1 | 0.46679 | 0.21628 | 0.30817 |
| 227 | O2 | 0.36666 | 0.16905 | 0.86642 |
| 228 | O3 | 0.41941 | 0.72702 | 0.34828 |
| 229 | O4 | 0.43249 | 0.59879 | 0.88366 |
| 230 | O5 | 0.87571 | 0.64966 | 0.36629 |
| 231 | O6 | 0.97558 | 0.71247 | 0.07536 |
| 232 | O7 | 0.92752 | 0.125 | 0.35804 |
| 233 | O8 | 0.90713 | 0.25553 | 0.69209 |
| 234 | S1 | 0.49134 | 0.16407 | 0.79895 |
| 235 | S2 | 0.34171 | 0.22022 | 0.42272 |
| 236 | S3 | 0.51274 | 0.69745 | 0.71817 |
| 237 | S4 | 0.34117 | 0.62244 | 0.48782 |
| 238 | S5 | 0.87012 | 0.76217 | 0.52144 |
| 239 | S6 | 0.98734 | 0.60088 | 0.13244 |
| 240 | S7 | 0.86723 | 0.11392 | 0.40774 |
| 241 | S8 | 0.00036 | 0.22186 | 0.78178 |

**Supplementary Table 2.** Atomic coordinates of the AA-stacking mode of DPP-Py-COF using DFTB+ method.

| Space group: P1 | | | | |
| --- | --- | --- | --- | --- |
| a = 52.6 Å, b = 37.6 Å and c = 4.3 Å. | | | | |
| α = 90°, β = 90°and γ = 90° | | | | |
| 1 | C1 | 0.56407 | 0.64004 | 0.0429 |
| 2 | C2 | 0.58644 | 0.62538 | 0.13092 |
| 3 | C3 | 0.60663 | 0.64952 | 0.13642 |
| 4 | C4 | 0.59421 | 0.68113 | 0.04151 |
| 5 | C5 | 0.5991 | 0.59385 | 0.03998 |
| 6 | C6 | 0.62924 | 0.63485 | 0.06067 |
| 7 | C7 | 0.5387 | 0.62293 | 0.00106 |
| 8 | C8 | 0.65454 | 0.65217 | 0.02322 |
| 9 | C9 | 0.55104 | 0.69834 | 0.79697 |
| 10 | C10 | 0.54794 | 0.7324 | 0.98028 |
| 11 | C11 | 0.51973 | 0.74239 | 0.93834 |
| 12 | C12 | 0.51321 | 0.77447 | 0.1428 |
| 13 | C13 | 0.48505 | 0.78271 | 0.08632 |
| 14 | C14 | 0.47967 | 0.81804 | 0.22187 |
| 15 | C15 | 0.6431 | 0.57664 | 0.82309 |
| 16 | C16 | 0.64507 | 0.54198 | 0.99753 |
| 17 | C17 | 0.6731 | 0.53092 | 0.96494 |
| 18 | C18 | 0.67836 | 0.4978 | 0.15775 |
| 19 | C19 | 0.70639 | 0.48849 | 0.10679 |
| 20 | C20 | 0.71067 | 0.45251 | 0.22983 |
| 21 | C21 | 0.56558 | 0.7573 | 0.78482 |
| 22 | C22 | 0.57017 | 0.78923 | 0.00253 |
| 23 | C23 | 0.62742 | 0.5179 | 0.79146 |
| 24 | C24 | 0.62046 | 0.48712 | 0.01593 |
| 25 | C25 | 0.32084 | 0.46495 | 0.00409 |
| 26 | C26 | 0.32123 | 0.42915 | 0.02055 |
| 27 | C27 | 0.34482 | 0.41152 | 0.99139 |
| 28 | C28 | 0.36822 | 0.42962 | 0.95765 |
| 29 | C29 | 0.36828 | 0.46546 | 0.96909 |
| 30 | C30 | 0.34446 | 0.4822 | 0.98625 |
| 31 | C31 | 0.34504 | 0.37554 | 0.99706 |
| 32 | C32 | 0.36866 | 0.35793 | 0.97031 |
| 33 | C33 | 0.39126 | 0.3764 | 0.91745 |
| 34 | C34 | 0.39101 | 0.41134 | 0.91121 |
| 35 | C35 | 0.29861 | 0.41047 | 0.0695 |
| 36 | C36 | 0.29879 | 0.37545 | 0.07353 |
| 37 | C37 | 0.32166 | 0.35734 | 0.03039 |
| 38 | C38 | 0.32186 | 0.32153 | 0.02551 |
| 39 | C39 | 0.3457 | 0.30501 | 0.0129 |
| 40 | C40 | 0.36912 | 0.32227 | 0.99308 |
| 41 | C41 | 0.29641 | 0.48512 | 0.00473 |
| 42 | C42 | 0.39252 | 0.48621 | 0.97325 |
| 43 | C43 | 0.29816 | 0.30004 | 0.03123 |
| 44 | C44 | 0.39337 | 0.30212 | 0.00208 |
| 45 | C45 | 0.29636 | 0.51909 | 0.11452 |
| 46 | C46 | 0.27337 | 0.53766 | 0.12356 |
| 47 | C47 | 0.25003 | 0.52264 | 0.02288 |
| 48 | C48 | 0.25006 | 0.48908 | 0.90355 |
| 49 | C49 | 0.27296 | 0.47078 | 0.89299 |
| 50 | C50 | 0.41631 | 0.47175 | 0.06669 |
| 51 | C51 | 0.43884 | 0.49075 | 0.07633 |
| 52 | C52 | 0.43829 | 0.52506 | 0.99683 |
| 53 | C53 | 0.41468 | 0.54037 | 0.90763 |
| 54 | C54 | 0.392 | 0.52103 | 0.89668 |
| 55 | C55 | 0.27386 | 0.31281 | 0.93477 |
| 56 | C56 | 0.25215 | 0.29201 | 0.92972 |
| 57 | C57 | 0.25436 | 0.25773 | 0.0187 |
| 58 | C58 | 0.27845 | 0.24469 | 0.11265 |
| 59 | C59 | 0.3001 | 0.2656 | 0.11847 |
| 60 | C60 | 0.39359 | 0.26837 | 0.88483 |
| 61 | C61 | 0.41649 | 0.24975 | 0.8847 |
| 62 | C62 | 0.43947 | 0.26456 | 0.00242 |
| 63 | C63 | 0.43918 | 0.29781 | 0.13167 |
| 64 | C64 | 0.41636 | 0.31618 | 0.13337 |
| 65 | C65 | 0.51609 | 0.64205 | 0.96407 |
| 66 | C66 | 0.49192 | 0.62652 | 0.94843 |
| 67 | C67 | 0.48995 | 0.59146 | 0.97232 |
| 68 | C68 | 0.51239 | 0.57211 | 0.01082 |
| 69 | C69 | 0.53649 | 0.58776 | 0.02628 |
| 70 | C70 | 0.67762 | 0.63385 | 0.04925 |
| 71 | C71 | 0.70151 | 0.65029 | 0.03742 |
| 72 | C72 | 0.70266 | 0.68538 | 0.00037 |
| 73 | C73 | 0.67969 | 0.70377 | 0.9741 |
| 74 | C74 | 0.65596 | 0.68728 | 0.98663 |
| 75 | C75 | 0.46444 | 0.57542 | 0.95875 |
| 76 | C76 | 0.72769 | 0.70278 | 0.00289 |
| 77 | C77 | 0.81783 | 0.96164 | 0.01892 |
| 78 | C78 | 0.81853 | 0.92585 | 0.02179 |
| 79 | C79 | 0.84246 | 0.90864 | 0.00392 |
| 80 | C80 | 0.86581 | 0.92729 | 0.9907 |
| 81 | C81 | 0.86518 | 0.9631 | 0.99262 |
| 82 | C82 | 0.84118 | 0.97902 | 0.00565 |
| 83 | C83 | 0.843 | 0.8726 | 0.99879 |
| 84 | C84 | 0.8669 | 0.85536 | 0.98292 |
| 85 | C85 | 0.8895 | 0.87458 | 0.9687 |
| 86 | C86 | 0.88903 | 0.90962 | 0.97142 |
| 87 | C87 | 0.79597 | 0.90678 | 0.04457 |
| 88 | C88 | 0.79651 | 0.87183 | 0.03956 |
| 89 | C89 | 0.81964 | 0.85404 | 0.011 |
| 90 | C90 | 0.82 | 0.81821 | 0.99893 |
| 91 | C91 | 0.84398 | 0.80204 | 0.98901 |
| 92 | C92 | 0.86751 | 0.81943 | 0.98334 |
| 93 | C93 | 0.79369 | 0.98241 | 0.02358 |
| 94 | C94 | 0.88835 | 0.98561 | 0.97937 |
| 95 | C95 | 0.79625 | 0.79647 | 0.99391 |
| 96 | C96 | 0.89178 | 0.79872 | 0.97938 |
| 97 | C97 | 0.79517 | 0.0177 | 0.0637 |
| 98 | C98 | 0.77294 | 0.03762 | 0.06388 |
| 99 | C99 | 0.74875 | 0.02264 | 0.02444 |
| 100 | C100 | 0.74705 | 0.98757 | 0.98182 |
| 101 | C101 | 0.76925 | 0.96787 | 0.98029 |
| 102 | C102 | 0.91321 | 0.97426 | 0.06786 |
| 103 | C103 | 0.93418 | 0.99647 | 0.06741 |
| 104 | C104 | 0.93064 | 0.03066 | 0.97991 |
| 105 | C105 | 0.90603 | 0.04213 | 0.89092 |
| 106 | C106 | 0.88518 | 0.01991 | 0.89101 |
| 107 | C107 | 0.77177 | 0.80994 | 0.92031 |
| 108 | C108 | 0.74986 | 0.78962 | 0.91433 |
| 109 | C109 | 0.75181 | 0.75505 | 0.97803 |
| 110 | C110 | 0.77609 | 0.7409 | 0.04831 |
| 111 | C111 | 0.79806 | 0.76147 | 0.05563 |
| 112 | C112 | 0.8906 | 0.76322 | 0.99414 |
| 113 | C113 | 0.91309 | 0.74364 | 0.99275 |
| 114 | C114 | 0.93725 | 0.75917 | 0.97612 |
| 115 | C115 | 0.93861 | 0.79444 | 0.96184 |
| 116 | C116 | 0.91622 | 0.81374 | 0.96348 |
| 117 | C117 | 0.07766 | 0.67776 | 0.97305 |
| 118 | C118 | 0.08072 | 0.64469 | 0.92731 |
| 119 | C119 | 0.10647 | 0.6354 | 0.97969 |
| 120 | C120 | 0.11982 | 0.66647 | 0.06649 |
| 121 | C121 | 0.06662 | 0.61326 | 0.91226 |
| 122 | C122 | 0.10881 | 0.60199 | 0.99219 |
| 123 | C123 | 0.05297 | 0.69632 | 0.96919 |
| 124 | C124 | 0.13333 | 0.58303 | 0.02082 |
| 125 | C125 | 0.10594 | 0.72526 | 0.23499 |
| 126 | C126 | 0.12672 | 0.7466 | 0.05745 |
| 127 | C127 | 0.11766 | 0.78321 | 0.07165 |
| 128 | C128 | 0.13629 | 0.80724 | 0.88686 |
| 129 | C129 | 0.12645 | 0.84303 | 0.92433 |
| 130 | C130 | 0.14559 | 0.86722 | 0.75525 |
| 131 | C131 | 0.07712 | 0.55452 | 0.12835 |
| 132 | C132 | 0.05313 | 0.53753 | 0.97761 |
| 133 | C133 | 0.05746 | 0.49961 | 0.00319 |
| 134 | C134 | 0.03622 | 0.47969 | 0.81811 |
| 135 | C135 | 0.03777 | 0.44327 | 0.9395 |
| 136 | C136 | 0.01445 | 0.42422 | 0.80197 |
| 137 | C137 | 0.15148 | 0.74206 | 0.27385 |
| 138 | C138 | 0.1752 | 0.74791 | 0.04215 |
| 139 | C139 | 0.03007 | 0.54775 | 0.20919 |
| 140 | C140 | 0.00494 | 0.54397 | 0.00289 |
| 141 | C141 | 0.05303 | 0.73141 | 0.92817 |
| 142 | C142 | 0.02997 | 0.7494 | 0.92256 |
| 143 | C143 | 0.0065 | 0.73251 | 0.95593 |
| 144 | C144 | 0.00616 | 0.69737 | 0.99403 |
| 145 | C145 | 0.02932 | 0.67943 | 0.00033 |
| 146 | C146 | 0.13358 | 0.5481 | 0.96371 |
| 147 | C147 | 0.15678 | 0.53026 | 0.96237 |
| 148 | C148 | 0.18011 | 0.54713 | 0.01619 |
| 149 | C149 | 0.18008 | 0.58202 | 0.07404 |
| 150 | C150 | 0.15683 | 0.59978 | 0.07516 |
| 151 | C151 | 0.98283 | 0.75247 | 0.95104 |
| 152 | C152 | 0.61033 | 0.10924 | 0.02391 |
| 153 | C153 | 0.60825 | 0.14276 | 0.02803 |
| 154 | C154 | 0.58233 | 0.15236 | 0.01954 |
| 155 | C155 | 0.56783 | 0.12136 | 0.00351 |
| 156 | C156 | 0.62248 | 0.17398 | 0.98128 |
| 157 | C157 | 0.58003 | 0.1855 | 0.9813 |
| 158 | C158 | 0.63457 | 0.08963 | 0.02057 |
| 159 | C159 | 0.55563 | 0.20457 | 0.98242 |
| 160 | C160 | 0.57896 | 0.06145 | 0.85432 |
| 161 | C161 | 0.55951 | 0.04158 | 0.07829 |
| 162 | C162 | 0.53267 | 0.04878 | 0.93146 |
| 163 | C163 | 0.51181 | 0.03213 | 0.15741 |
| 164 | C164 | 0.48582 | 0.03827 | 0.98313 |
| 165 | C165 | 0.4717 | 0.00501 | 0.94544 |
| 166 | C166 | 0.61086 | 0.22889 | 0.71507 |
| 167 | C167 | 0.6147 | 0.2615 | 0.92153 |
| 168 | C168 | 0.64323 | 0.27042 | 0.89869 |
| 169 | C169 | 0.64955 | 0.30202 | 0.11348 |
| 170 | C170 | 0.67563 | 0.3154 | 0.99961 |
| 171 | C171 | 0.67907 | 0.35069 | 0.14978 |
| 172 | C172 | 0.56512 | 0.00398 | 0.04014 |
| 173 | C173 | 0.59007 | 0.99437 | 0.22821 |
| 174 | C174 | 0.59847 | 0.28791 | 0.72679 |
| 175 | C175 | 0.59207 | 0.31793 | 0.96459 |
| 176 | C176 | 0.63367 | 0.05446 | 0.0489 |
| 177 | C177 | 0.65621 | 0.03541 | 0.0484 |
| 178 | C178 | 0.68008 | 0.05134 | 0.02224 |
| 179 | C179 | 0.68133 | 0.08658 | 0.99797 |
| 180 | C180 | 0.65864 | 0.10558 | 0.99762 |
| 181 | C181 | 0.55548 | 0.23853 | 0.08586 |
| 182 | C182 | 0.53236 | 0.25644 | 0.1027 |
| 183 | C183 | 0.50913 | 0.24045 | 0.01812 |
| 184 | C184 | 0.50912 | 0.20647 | 0.91557 |
| 185 | C185 | 0.53226 | 0.1887 | 0.89882 |
| 186 | C186 | 0.70323 | 0.03042 | 0.02217 |
| 187 | C187 | 0.48518 | 0.25961 | 0.04438 |
| 188 | C188 | 0.20414 | 0.52781 | 0.99728 |
| 189 | C189 | 0.05871 | 0.14116 | 0.03753 |
| 190 | C190 | 0.08303 | 0.13359 | 0.95728 |
| 191 | C191 | 0.09895 | 0.16199 | 0.99578 |
| 192 | C192 | 0.08211 | 0.18877 | 0.11501 |
| 193 | C193 | 0.10015 | 0.10651 | 0.85247 |
| 194 | C194 | 0.12343 | 0.15418 | 0.93212 |
| 195 | C195 | 0.03699 | 0.11711 | 0.04754 |
| 196 | C196 | 0.14547 | 0.1777 | 0.94517 |
| 197 | C197 | 0.03553 | 0.19313 | 0.3099 |
| 198 | C198 | 0.02861 | 0.22617 | 0.12401 |
| 199 | C199 | 0.99939 | 0.23005 | 0.13612 |
| 200 | C200 | 0.99039 | 0.26187 | 0.94429 |
| 201 | C201 | 0.9613 | 0.26412 | 0.95889 |
| 202 | C202 | 0.95292 | 0.29894 | 0.83817 |
| 203 | C203 | 0.14702 | 0.10217 | 0.67353 |
| 204 | C204 | 0.15497 | 0.0705 | 0.87676 |
| 205 | C205 | 0.18433 | 0.06907 | 0.86928 |
| 206 | C206 | 0.19496 | 0.03981 | 0.08854 |
| 207 | C207 | 0.22409 | 0.04032 | 0.07355 |
| 208 | C208 | 0.23454 | 0.00777 | 0.23005 |
| 209 | C209 | 0.0418 | 0.25483 | 0.32705 |
| 210 | C210 | 0.04677 | 0.28505 | 0.08483 |
| 211 | C211 | 0.14313 | 0.04034 | 0.68113 |
| 212 | C212 | 0.14177 | 0.00917 | 0.91939 |
| 213 | C213 | 0.01165 | 0.12823 | 0.9931 |
| 214 | C214 | 0.99119 | 0.10525 | 0.99881 |
| 215 | C215 | 0.99591 | 0.07088 | 0.06106 |
| 216 | C216 | 0.02113 | 0.05979 | 0.11712 |
| 217 | C217 | 0.04152 | 0.08273 | 0.10965 |
| 218 | C218 | 0.17047 | 0.16547 | 0.0012 |
| 219 | C219 | 0.19144 | 0.18754 | 0.00304 |
| 220 | C220 | 0.18763 | 0.22216 | 0.94747 |
| 221 | C221 | 0.16271 | 0.23451 | 0.89383 |
| 222 | C222 | 0.14176 | 0.2124 | 0.89307 |
| 223 | C223 | 0.97502 | 0.04597 | 0.06133 |
| 224 | C224 | 0.20931 | 0.24582 | 0.9475 |
| 225 | N1 | 0.56835 | 0.67581 | 0.99442 |
| 226 | N2 | 0.62513 | 0.59905 | 0.01072 |
| 227 | N3 | 0.22655 | 0.54161 | 0.04675 |
| 228 | N4 | 0.23255 | 0.23567 | 0.01557 |
| 229 | N5 | 0.46212 | 0.5432 | 0.00698 |
| 230 | N6 | 0.95152 | 0.0543 | 0.98326 |
| 231 | N7 | 0.72615 | 0.04332 | 0.02761 |
| 232 | N8 | 0.7287 | 0.73535 | 0.97228 |
| 233 | N9 | 0.10247 | 0.69257 | 0.05769 |
| 234 | N10 | 0.08352 | 0.58677 | 0.9552 |
| 235 | N11 | 0.96015 | 0.73898 | 0.97637 |
| 236 | N12 | 0.58467 | 0.09472 | 0.00595 |
| 237 | N13 | 0.6054 | 0.20032 | 0.95158 |
| 238 | N14 | 0.46299 | 0.2459 | 0.97865 |
| 239 | N15 | 0.05742 | 0.1765 | 0.13629 |
| 240 | N16 | 0.12488 | 0.11874 | 0.8403 |
| 241 | O1 | 0.6049 | 0.708 | 0.98804 |
| 242 | O2 | 0.58846 | 0.56718 | 0.97497 |
| 243 | O3 | 0.14209 | 0.6691 | 0.16634 |
| 244 | O4 | 0.04341 | 0.61039 | 0.8805 |
| 245 | O5 | 0.5447 | 0.11889 | 0.95653 |
| 246 | O6 | 0.6456 | 0.17668 | 0.93754 |
| 247 | O7 | 0.08866 | 0.21712 | 0.20247 |
| 248 | O8 | 0.09387 | 0.07792 | 0.77026 |

**Supplementary Table 3.** Selective photocatalytic coupling of benzylamine to N-benzylidenebenzylamine over different catalysts.

| Catalyst | Time  (h) | Substrate | product | Con.  (%) | Sel.  (%) | Ref. |
| --- | --- | --- | --- | --- | --- | --- |
| PyTTA-T PA COFs /Pt NC | 0.33 | Benzylamine | N-benzylidenebenzylamine | 99 | 98 | This work |
| ClBD-BTT | 6 | Benzylamine | N-benzylidenebenzylamine | 82 | ‒ | 3 |
| Tp-BTD-25 | 20 | Benzylamine | N-benzylidenebenzylamine | 94 | 98 | 4 |
| NH2-MIL-125(Ti) | 12 | Benzylamine | N-benzylidenebenzylamine | 73 | 86 | 5 |
| TiO_2_ | 9 | Benzylamine | N-benzylidenebenzylamine | 99 | 85 | 6 |
| COF-LZU1 | 8 | Benzylamine | N-benzylidenebenzylamine | 23 | 99 | 7 |
| Th6-C8A | 16 | Benzylamine | N-benzylidenebenzylamine | 97 | 99 | 8 |
| PyTTA-TPA COFs /Pt NC | 3 | Benzylamine | N-benzylidenebenzylamine | 98 | 97 | 9 |
| TFB-33-DMTH | 20 | Benzylamine | N-benzylidenebenzylamine | 99 | 100 | 10 |
| Cu_0.5_/NS-OV | 12 | Benzylamine | N-benzylidenebenzylamine | 40.1 | 88.9 | 11 |
| Pd_0.5_/NS-OV | 12 | Benzylamine | N-benzylidenebenzylamine | 95.1 | 98.2 | 11 |
| Pt_0.5_/NS-OV | 12 | Benzylamine | N-benzylidenebenzylamine | 85.9 | 90.7 | 11 |
| Pd/NH2-MIL-125 | 12 | Benzylamine | N-benzylidenebenzylamine | 80.5 | 85.5 | 12 |

**Supplementary Table 4.** Selective photocatalysis coupling of benzylamine to N-benzylidenebenzylamine on TpDPP-Py COFs film.

|  | | | | | |
| --- | --- | --- | --- | --- | --- |
| Number | Catalyst | hv | Atmosphere | Conversion rate/ | Selectivity/ % |
| 1 | ‒ | ‒ | Ar | ‒ | ‒ |
| 2 | ‒ | + | Ar | ‒ | ‒ |
| 3 | ‒ | ‒ | N_2_ | ‒ | ‒ |
| 4 | ‒ | + | N_2_ | ‒ | ‒ |
| 5 | ‒ | ‒ | O_2_ | ‒ | ‒ |
| 6 | ‒ | + | O_2_ | 0.5% | 96.6% |
| 7 | + | ‒ | Ar | ‒ | ‒ |
| 8 | + | + | Ar | 5.1% | 97.5% |
| 9 | + | ‒ | N_2_ | ‒ | ‒ |
| 10 | + | + | N_2_ | 3.5% | 97.5% |
| 11 | + | ‒ | O_2_ | ‒ | ‒ |
| 12 | + | + | O_2_ | 99% | 98% |

**Supplementary References**

1. Kang S. H., Lee D., Kim H., Choi W., Oh J., J. Oh H., Yang C. Effects of the polarity and bulkiness of end-functionalized side chains on the charge transport of dicyanovinyl-end-capped diketopyrrolopyrrole-based n‑type small molecules. *ACS Appl. Mater. Interfaces.* **13**, 52840–52849 (2021).

2. J. Xu, W. Tang, C. Yang, I. Manke, N. Chen, F. Lai, T. Xu, S. An, H. Liu, Z. Zhang, Y. Cao, N. Wang, S. Zhao, D. Niu, R. Chen. A Highly Conductive COF@CNT Electrocatalyst Boosting Polysulfide Conversion for Li−S Chemistry. *ACS Energy Lett*, **6**, 3053−3062 (2021).

3. Chu C., Qin Y., Ni C., Zou J. Halogenated benzothiadiazole-based conjugated polymers as efficient photocatalysts for dye degradation and oxidative coupling of benzylamines. *Chinese Chem. Lett.* **33**, 2736−2740 (2022).

4. Li X., Yang S., Zhang F., Zheng L., Lang X. Facile synthesis of 2D covalent organic frameworks for cooperative photocatalysis with TEMPO: The selective aerobic oxidation of benzyl amines. *Appl. Catal. B-Environ.* **303**, 120846 (2022).

5. Sun D., Ye L., Li Z. Visible-light-assisted aerobic photocatalytic oxidation of amines to imines over NH2-MIL-125(Ti). *Appl. Catal. B-Environ.* **164**, 428−432 (2015).

6. Xu C., Liu H., Li D., Su J-H., Jiang H-L. Direct evidence of charge separation in a metal–organic framework: efficient and selective photocatalytic oxidative coupling of amines *via* charge and energy transfer. *Chem. Sci.* **9**, 3152−3158 (2018).

7. Wu Z., Huang X., Li X., Hai G., Li B., Wang G. Covalent-organic frameworks with keto-enol tautomerism for efficient photocatalytic oxidative coupling of amines to imines under visible light. *Sci. China Chem.* **64**, 2169−2179 (2021).

8. Niu Q., Huang Q., Yu T., Liu J., Shi J., Dong L., Li S., Lan Y. Achieving high photo/thermocatalytic product selectivity and conversion *via* thorium clusters with switchable functional ligands. *J. Am. Chem. Soc.* **144**, 18586−18594 (2022).

9. Liu, Y., Tan, H., Sun, J., Wei, Y., Liu, M., Hong, J., Shang, S., Wang, X., Li, L., Gu, Y., Ye, N., Chen, J., Yang, Y., Guo, S. and Liu, Y. A Universal room-temperature approach to large-area continuous COFs film for photocatalytic coupling of amines. *Adv. Funct. Mater.* **33**, 2302874 (2023).

10. Liu S., Su Q., Qi W., Luo K., Sun X., Ren H., Wu Q. Highly hydrophilic covalent organic frameworks as efficient and reusable photocatalysts for oxidative coupling of amines in aqueous solution. *Catal. Sci. Technol.* **12**, 2837−2845 (2022).

11. Wei S., Zhong H., Wang H., Song Y., Jia C., Anpo M., Wu L. Oxygen vacancy enhanced visible light photocatalytic selective oxidation of benzylamine over ultrathin Pd/BiOCl nanosheets. *Appl. Catal. B-Environ.* **305**, 121032 (2022).

12. Wang H., Yu J., Wei S., Lin M., Song Y., Wu L. Surface coordination enhanced visible-light photocatalytic coupling of benzylamine to N-benzylidene benzylamine over the Pd/NH2-MIL-125(Ti) nanosheets. *Chem. Eng. J.* **441**, 136020 (2022).
